# Histologic and Histomorphometric Evaluation of Bone Regeneration Using Human Allogeneic Bone Graft with or Without Mesenchymal Stem Cell–Conditioned Media in a Rabbit Calvarial Defect Model

**DOI:** 10.3390/jfb16070251

**Published:** 2025-07-07

**Authors:** Hyung-Gyun Kim, Yong-Suk Moon, Dong-Seok Sohn

**Affiliations:** 1 Department of Dentistry and Advanced General Dentistry, Daegu Catholic University School of Medicine, Daegu 42472, Republic of Korea; hgkim25@cu.ac.kr; 2 Department of Anatomy, Daegu Catholic University School of Medicine, Daegu 42472, Republic of Korea; ysmoon@cu.ac.kr; 3 Department of Dentistry and Oral and Maxillofacial Surgery, Daegu Catholic University School of Medicine, Daegu 42472, Republic of Korea

**Keywords:** bone regeneration, mesenchymal stem cell–conditioned media, human allograft bone

## Abstract

Alveolar bone loss due to trauma, extraction, or periodontal disease often requires bone grafting prior to implant placement. Although human allograft bone is widely used as an alternative to autograft, it has limited osteoinductive potential and a prolonged healing time. Mesenchymal stem cell–conditioned media (MSC-CM), rich in paracrine factors, has emerged as a promising adjunct to enhance bone regeneration. This study evaluated the regenerative effect of MSC-CM combined with human allograft bone in a rabbit calvarial defect model. Bilateral 8 mm defects were created in eight rabbits. Each animal received a human allograft alone (HB group) on one side and an allograft mixed with MSC-CM (HB+GF group) on the other. Histological and histomorphometric analyses were performed at 2 and 8 weeks postoperatively. Both groups showed new bone formation, but the HB+GF group demonstrated significantly greater bone regeneration at both time points (*p* < 0.05). New bone extended into the defect center in the HB+GF group. Additionally, greater graft resorption and marrow formation were observed in this group at 8 weeks. These findings suggest that MSC-CM enhances the osteogenic performance of human allograft bone and may serve as a biologically active adjunct for bone regeneration.

## 1. Introduction

Alveolar bone deficiency due to resorption and defects is caused by various factors such as trauma, tooth extraction, and periodontal disease [1,2,3]. This deficiency reduces the width and height of the alveolar ridge, making it challenging to place intraosseous implants and perform prosthetic reconstruction treatment [2]. To address this issue, bone grafting for bone regeneration is widely used [3].

While the precise mechanisms regulating bone regeneration at the molecular level are not fully understood, the regenerative mechanisms of bone graft materials can be classified into three types: osteogenesis, osteoinduction, and osteoconduction. Osteogenesis is the process of bone formation mediated by osteoblasts. Osteoinduction refers to the process by which mesenchymal stem cells are stimulated to differentiate into osteoblasts, thereby initiating new bone formation. Osteoconduction functions as a scaffold for new bone formation, facilitating the integration of surrounding bone to generate new bone [4].

Autogenous bone grafts possess all three mechanisms of bone regeneration, demonstrate excellent osteogenic potential, and are widely regarded as the gold standard graft material due to their immunocompatibility, which prevents inflammatory or immune responses [5,6,7]. However, their clinical application is hindered by the need for additional donor sites, challenges in harvesting sufficient quantities, and potential complications such as root resorption [3,4].

Human allogeneic bone graft materials are commonly used in clinical practice as substitutes for autogenous bone. Although they share some characteristics with autografts, allogeneic grafts are acellular, since bone-forming cells are eliminated during tissue processing through decalcification and freeze-drying. Compared with xenografts or synthetic substitutes, they are more biologically similar to native human tissue, do not require a donor site, and can be used in larger quantities when needed [8,9]. However, these materials are associated with certain limitations, such as the risk of infection, low osteoinductive potential, a prolonged healing period for bone regeneration, and the potential for immune responses due to residual antigens that may persist even after processing [10].

To overcome these limitations, extensive research has focused on regenerative medicine, which aims to restore and stabilize normal tissue or organ function through various approaches, including tissue engineering, gene therapy, cell therapy, and growth factor-based therapy [11]. Among these, mesenchymal stem cells (MSCs) are considered promising due to their relative ease of isolation and ex vivo expansion and their ability to promote tissue repair through the paracrine secretion of various growth factors [12,13,14,15]. However, several challenges remain, including high treatment costs, safety concerns, and the invasiveness associated with harvesting bone marrow from patients. Furthermore, recent studies have reported limited long-term survival of transplanted MSCs [16,17,18].

Research has been conducted on conditioned media derived from MSC cultures (MSC-CM) to address these issues. MSC-CM contains a variety of accumulated paracrine factors secreted by MSCs during cell culture and is reported to play a beneficial role in tissue regeneration [15,17]. Numerous studies have investigated tissue regeneration using MSC-CM, including alveolar bone regeneration. Recent preclinical and clinical studies have demonstrated the potential of mesenchymal stem cell–conditioned media (MSC-CM) in bone regeneration. MSC-CM has been shown to enhance osteogenic differentiation and promote bone healing in various animal models, such as calvarial defects and peri-implant bone regeneration. Clinical applications have also been explored, including its use in sinus floor augmentation and periodontal tissue regeneration. These studies commonly employed MSC-CM alone or in combination with synthetic bone substitutes [19,20,21,22,23]. To our knowledge, this is the first study to evaluate the regenerative effect of MSC-CM combined with human allogeneic bone in a rabbit calvarial defect model, representing a more clinically relevant application than previous studies using synthetic materials.

In this study, human allogenic bone graft material mixed with conditioned media derived from mesenchymal stem cells and human allogenic bone graft material alone were grafted into bone defects created in the rabbit skull. The effect of conditioned media on the healing process and bone regeneration of human allogenic bone was compared and analyzed histologically and histomorphometrically.

## 2. Materials and Methods

### 2.1. Experimental Animals

Eight adult male New Zealand White rabbits, each weighing approximately 2.8 to 3.2 kg (average weight: 3.0 kg), were included in this study. Ethical clearance for the animal investigation was granted by the Institutional Animal Care and Use Committee (IACUC) of Daegu Catholic University Medical Center (protocol code DCIAFCR-210623-07-Y and date of approval: 23 June 2021).

### 2.2. Experimental Materials

In this study, human allogeneic bone graft material (DO BONE^®^, RENEW-MEDICAL Co., Ltd., Bucheno-si, Gyeonggi-do, Republic of Korea) and human mesenchymal stem cell–conditioned media (Hucord Co., Ltd., Yonging-si, Gyeonggi-do, Republic of Korea) were used.

### 2.3. Experimental Group Settings

**Figure 1:**
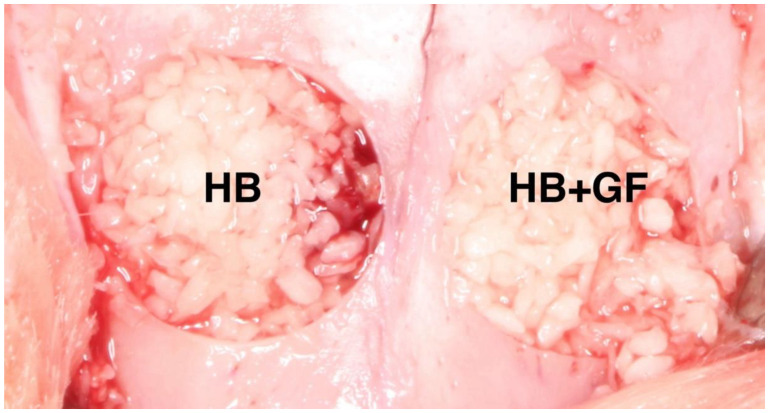
Application of bone materials. HB—human bone graft material particles, HB+GF—human bone graft material mixed with human cord blood stem cell conditioned media.

General anesthesia was induced by an intramuscular injection of 30 mg/kg ketamine (ketamine hydrochloride, Yuhan Corporation, Seoul, Republic of Korea) and 10 mg/kg xylazine (Rompun, Bayer Korea, Seoul, Republic of Korea). After confirming that general anesthesia had been achieved, the hair on the cranial region was removed, the surgical area was disinfected with povidone-iodine, and 2% lidocaine (Lidocaine HCl, Huons, Seoul, Republic of Korea) was injected subcutaneously along the midline of the cranial region to induce infiltration anesthesia. Each rabbit was placed on the operating table, an incision was made along the center of the frontal bone from front to back, and the full-thickness flap was lifted to expose the frontal bone. Then, using an 8 mm diameter trephine burr, we excised bone on both sides of the midline of each rabbit’s frontal bone. The resected bone was removed carefully to avoid damaging the underlying tissue. One of the two bone defect areas was filled with 0.5 cc of particle-type human allogenic bone graft material alone (HB group). The other area was filled with 0.5 cc of particle-type human allogenic bone graft material mixed with 0.2 cc human mesenchymal stem cell–conditioned media (HB+GF group) (Figure 1). The surgical site was closed using 4-0 monofilament nylon (Blue Nylon, Ailee Co., Busan, Republic of Korea). Following the procedure, each rabbit received an intramuscular injection of gentamicin (20 mg/kg; Dong-hwa Co., Seoul, Republic of Korea) once daily for three consecutive days.

### 2.4. Tissue Preparation

Under general anesthesia, 4 out of 8 rabbits were sacrificed 2 weeks after surgery, and the remaining 4 were sacrificed 8 weeks after surgery. The bone graft site was dissected using a microtome, separated from the frontal bone, and then fixed in neutral buffered formalin (NBF) for 24 h. After fixation, it was rinsed with 0.1 M phosphate buffer and decalcified in 10% formic acid for 10 days. The specimen was embedded in paraffin (Paraplast^®^, Leica Biosystems, Deer Park, IL, USA), and 5 µm thick serial sections were sliced through the center of the circular bone defect and the subcutaneous area. The section specimens were stained using hematoxylin–eosin (H&E) and Masson’s trichrome (MT) staining, and the new bone and tissue changes at the cranial defect site were examined under an optical microscope. For each group, images were acquired from 10 randomly chosen areas using the AxioCam MRc5 digital camera (Carl Zeiss, Oberkochen, Baden-Württemberg, Germany), which was mounted on an Axiophot microscope (Carl Zeiss, Germany). Image analysis was performed with the AxioVision SE64 software package (Carl Zeiss, Germany). Histomorphometric measurements of the total augmented area, new bone area, graft material (human bone particle) area, soft tissue area, and bone marrow area were performed. The total augmented area included new bone, graft material, fibrous tissue, and vascular tissue within the defect. In both the HB and HB+GF groups, the proportion of new bone or remaining graft material was calculated by dividing its respective area by the total augmented area.

### 2.5. Growth Factor Profiling of MSC-CM by Multiplex Immunoassay

The MSC-CM used in this study was commercially obtained from Hucord Co., Ltd. (Yongin, Gyeonggi-do, Republic of Korea). The concentrations of specific growth factors in the CM, including IGF-1 and TGF-β1, were quantified using a multiplex bead-based immunoassay (Luminex system), and the data were provided by the manufacturer for research purposes.

### 2.6. Statistical Analysis

The data processing and statistical analysis were conducted using appropriately validated software (SPSS, version 25.0, SPSS Inc, Chicago, IL, USA). The statistical significance of differences within and between groups was assessed using one-way analysis of variance (ANOVA) with Tukey’s method. Quantitative results were presented as mean ± standard error, and a *p* value of <0.05 indicated statistical significance.

## 3. Results

### 3.1. Histological Analysis

In H-E and MT staining, human allogenic bone graft material was weakly stained but remained clearly distinguishable from adjacent tissue. In MT staining, host-derived lamellar bone appeared reddish, while woven or newly formed bone showed a bluish hue. When examined under an optical microscope following H-E and MT staining, no inflammatory response was observed in either the HB or HB+GF groups. In both groups, human allogenic bone graft material maintained space in the defect area, so the center of the defect did not collapse.

**Figure 2:**
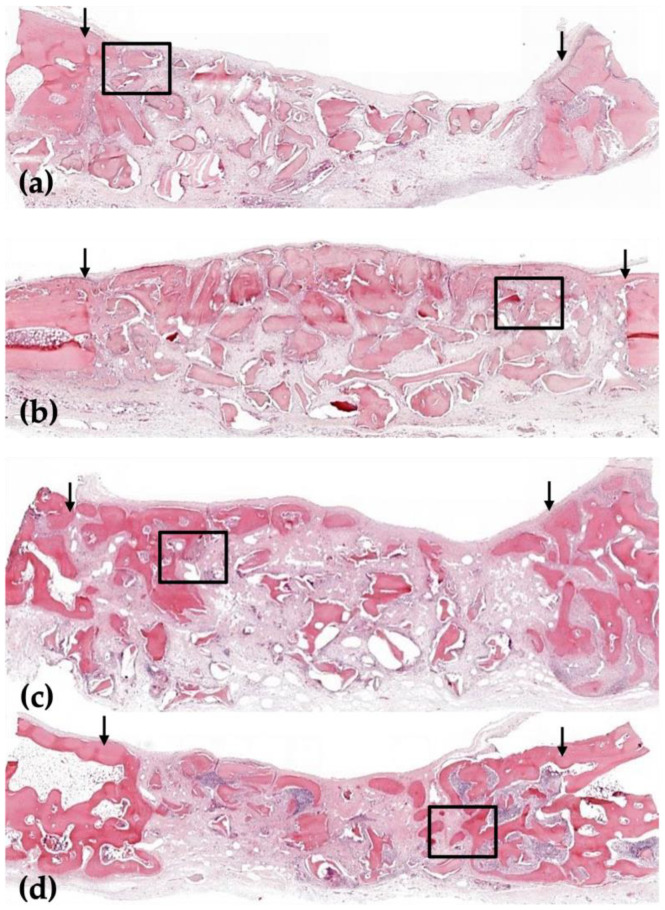
Low-magnification images of the rabbit calvaria after surgery in the 2-week HB group (**a**), in the 2-week HB+GF group (**b**), in the 8-week HB group (**c**), and in the 8-week HB+GF group (**d**). The arrows indicate the margins of the defect. The boxed areas are presented at higher magnification in Figure 4: hematoxylin–eosin stain (×20).

**Figure 3:**
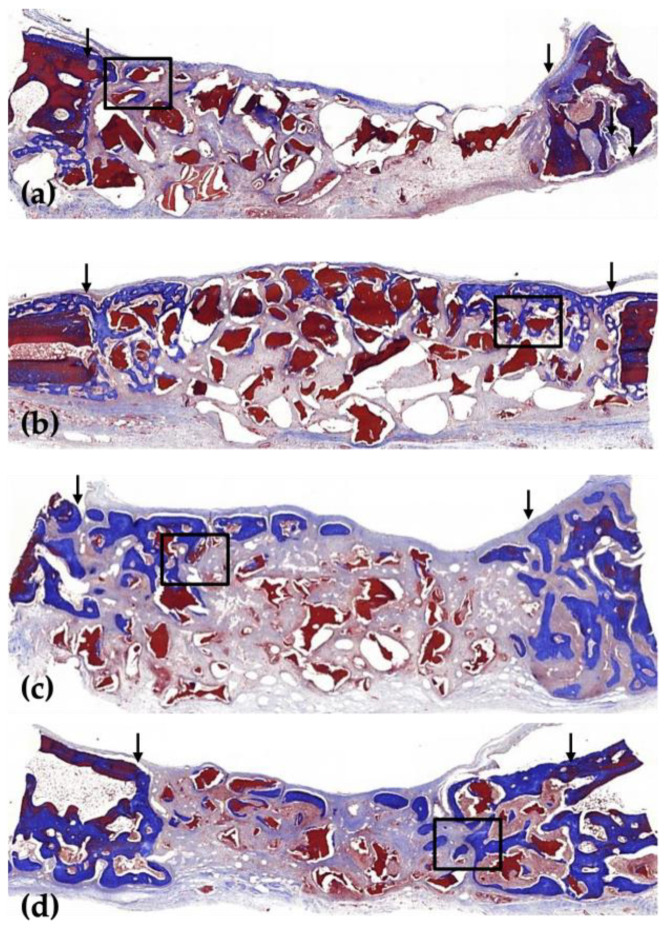
Low-magnification images of the rabbit calvaria after surgery in the 2-week HB group (**a**), in the 2-week HB+GF group (**b**), in the 8-week HB group (**c**), and in the 8-week HB+GF group (**d**). The arrows indicate the margins of the defect. The boxed areas are presented at a higher magnification in Figure 5. Masson’s trichrome stain (×20).

In the 2-week group, a small amount of new bone was formed in both groups, limited to the border of the defect area, but the amount of new bone was found to be different. In the 8-week group, a large amount of new bone formed around the border of the defect, and human allogenic bone particles were present in both groups (Figure 2 and Figure 3).

**Figure 4:**
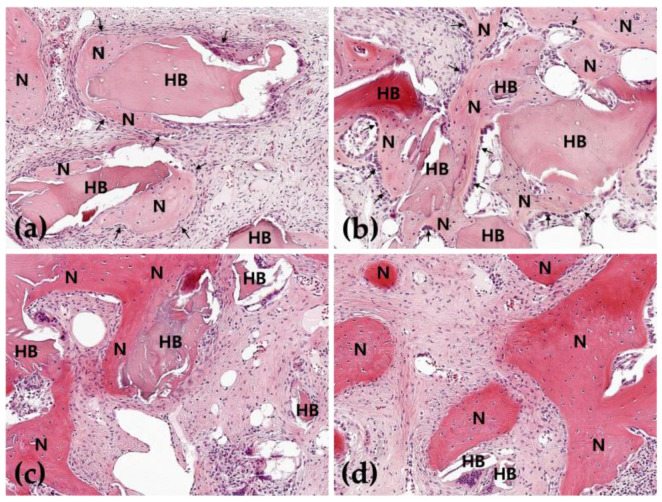
Photomicrograph showing the new bone formation after surgery in the 2-week HB group (**a**), in the 2-week HB+GF group (**b**), in the 8-week HB group (**c**), and in the 8-week HB+GF group (**d**). (**a**) New bone was identified along the surface of the human allogenic bone particles, with osteoblasts (arrows) localized on the surface of the new bone; (**b**) new bone was partially formed adjacent to the human allogenic bone particles and extended into the connective tissue between the human allogenic bone particles, many osteoblasts (arrows) were observed on the surface of new bone; (**c**) new bone on the surface of the human allogenic bone particles increased; (**d**) the thickness and the density of new bone were highly increased, the size of human allogenic bone particles was decreased, and the density of connective tissue was increased. N—new bone; HB—human allogenic bone particle; hematoxylin–eosin stain (×200).

**Figure 5:**
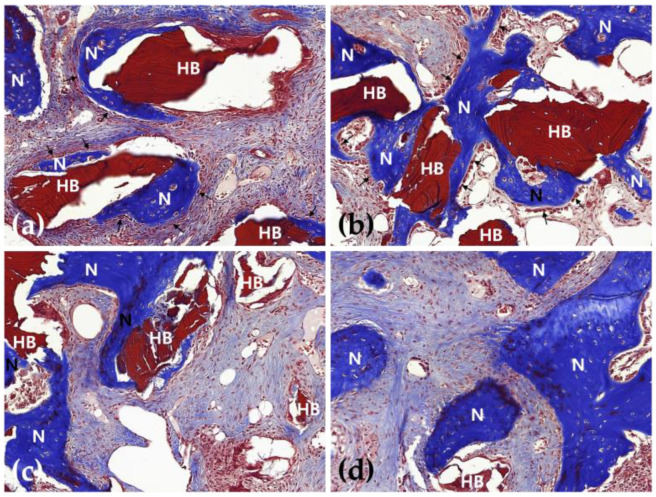
Photomicrograph showing the new bone formation after surgery in the 2-week HB group (**a**), in the 2-week HB+GF group (**b**), in the 8-week HB group (**c**), and in the 8-week HB+GF group (**d**). (**a**) New bone was identified along the surface of the human allogenic bone particles, with osteoblasts (arrows) localized on the surface of the new bone; (**b**) new bone was partially formed adjacent to the human allogenic bone particles and extended into the connective tissue between the human allogenic bone particles; (**c**) many osteoblasts (arrows) were observed on the surface of new bone (B), new bone on the surface of the human allogenic bone particles increased; (**d**) the thickness and the density of new bone were highly increased, the size of human allogenic bone particles was decreased, and the density of connective tissue was increased. N—new bone; HB—human allogenic bone particle; Masson’s trichrome stain (×200).

In the 2-week HB group, new bone was partially observed at the border of the defect but not at the center of the defect. New bone was observed on the outer surface of human allogenic bone particles, and osteoblasts were also observed on the outer surface of the new bone (Figure 2a, Figure 3a, Figure 4a and Figure 5a). In the 8-week HB group, compared with the 2-week HB group, the amount of new bone observed on the outer surface of human allogenic bone particles increased, a small amount of new bone was observed in the center of the defect, and the size and distribution density of human allogenic bone particles in the defect area decreased and combined with increasing tissue density (Figure 2c, Figure 3c, Figure 4c and Figure 5c).

In the 2-week HB+GF group, a small amount of new bone was observed at the border of the defect area and in the center. New bone was observed on the outer surface of human bone allogenic particles, extending into the connective tissue between human bone particles. Many osteoblasts were observed on the outer surface of the new bone (Figure 2b, Figure 3b, Figure 4b and Figure 5b). In the 8-week HB+GF group, compared with the 2-week HB+GF group, the size and distribution density of new bone in the defect area significantly increased, while the dimensions and spatial concentration of human allogenic bone particles were reduced, and the amount of connective tissue became more prominent. A small bone marrow space was also observed between the new bones (Figure 2d, Figure 3d, Figure 4d and Figure 5d).

### 3.2. Histomorphometric Analysis

**Figure 6:**
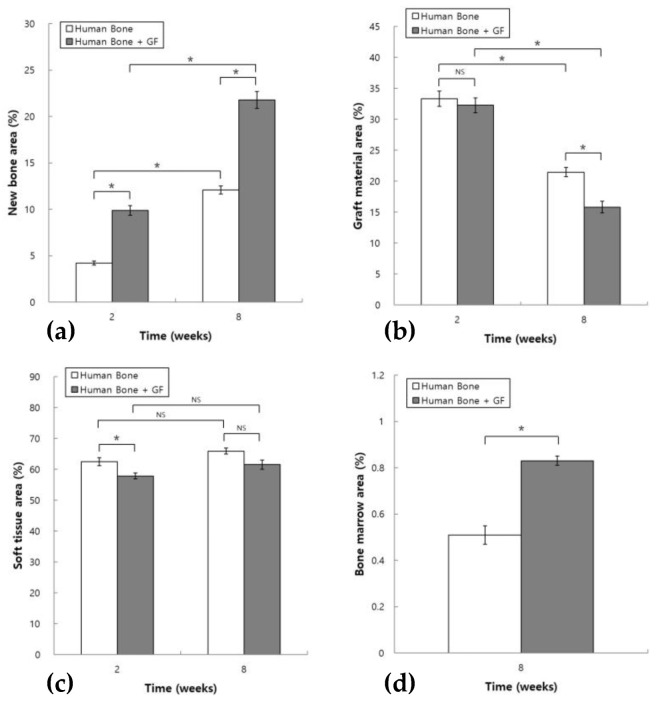
Histomorphometric measurement of tissue composition within the augmented area at 2 and 8 weeks: (**a**) new bone, (**b**) graft material, (**c**) soft tissue, and (**d**) bone marrow (8-week group only). (* *p* < 0.05, NS: not significant).

In the HB group, the ratio of new bone to the area of the augmented area in the 2- and 8-week groups was 4.21 ± 0.20 and 12.10 ± 0.44%, respectively. In the HB+GF group, the ratio of new bone within the augmented area was 9.89 ± 0.51% at 2 weeks and 21.79 ± 0.90% at 8 weeks. Statistical analysis using one-way ANOVA followed by post hoc testing revealed a significantly larger new bone area in the 8-week group compared with the 2-week group in both experimental groups, with *p* < 0.05. The new bone area significantly increased in the 2-week HB+GF group compared with the 2-week HB group, and the same result was seen in the 8-week group (Figure 6a).

In the HB group, the ratio of graft material within the augmented area was 33.34 ± 1.25% at 2 weeks and 21.46 ± 0.75% at 8 weeks. In the HB+GF group, the ratio of graft material within the augmented area was 32.27 ± 1.18% at 2 weeks and 15.83 ± 0.94% at 8 weeks. The graft material area in the 8-week group was significantly less than in the 2-week group in both groups, with *p* < 0.05. In the 2-week group, the graft material area in the HB+GF group was not significantly different from that in the HB group. In the 8-week group, the area of graft material decreased significantly in the HB+GF group, compared with the HB group, with *p* < 0.05 (Figure 6b).

In the HB group, the ratio of soft tissue within the augmented area was 62.45 ± 1.29% at 2 weeks and 65.93 ± 0.95% at 8 weeks. In the HB+GF group, the ratio of soft tissue within the augmented area was 57.84 ± 0.99% at 2 weeks and 61.55 ± 1.48% at 8 weeks. The soft tissue area in the 8-week group was not significantly different from that in the 2-week group, with *p* < 0.05. In the 2-week group, the soft tissue area decreased significantly in the HB+GF group compared with the HB group. In the 8-week group, the soft tissue area in the HB+GF group was not significantly different from that in the HB group (Figure 6c).

The ratio of bone marrow within the augmented area was 0.51 ± 0.04% in the 8-week HB group and 0.83 ± 0.02% in the 8-week HB+GF group. The area of bone marrow increased significantly in the 8-week HB+GF group compared with the 8-week HB group (Figure 6d).

### 3.3. Quantitative Results of Growth Factors in MSC-CM

Growth factor levels in the MSC-CM were reported based on multiplex immunoassay data provided by the manufacturer (Hucord Co., Ltd., Yongin, Republic of Korea). According to the data, the MSC-CM contained 119.5 pg/mL of IGF-1 and 3238.3 pg/mL of TGF-β1. Vascular endothelial growth factor (VEGF) was not detected.

While the composition report included additional bioactive factors, the present study focused on IGF-1, TGF-β1, and VEGF to allow for comparison with previous studies and to maintain a consistent analytical framework.

## 4. Discussion

Several studies have been conducted to regenerate damaged periodontal or bone tissue using various bone graft materials. Numerous animal experiments and clinical studies have been carried out to identify clinically suitable bone graft materials. Bone grafting materials for bone regeneration must initially demonstrate good biocompatibility and support the attachment and differentiation of bone cells. The grafting material particles need to be absorbed and replaced by new tissue or closely integrated with new bone [24]. However, to date, no bone graft material satisfies all these conditions [25], and they have several disadvantages. One of them is the prolonged healing period required before placing dental implants, which causes inconveniences such as delayed recovery for patients and a prolonged period of decline in masticatory function, which reduces the quality of life [20].

Many studies have been conducted on growth factors mediating the migration and differentiation of osteogenic cells and the extracellular matrix to achieve faster and more successful bone healing to address these disadvantages [26]. Bone morphogenetic protein-2 (BMP-2) and platelet-derived growth factor (PDGF) are commercially available growth factors. They are widely used in clinical practice due to their effectiveness in the regeneration of bone and periodontal tissues [27,28]. However, many studies have reported that achieving sufficient regeneration of bone and periodontal tissues using BMP-2 and PDGF requires high doses, which are associated with serious side effects such as severe inflammation and swelling [29,30]. Furthermore, when applied with only a single growth factor, the ability for bone regeneration becomes limited.

Research has been conducted to optimize bone regeneration by combining two or more growth factors to overcome these limitations. Additionally, studies in regenerative medicine employing the concept of tissue regeneration using stem cells have been actively pursued [31,32]. Mesenchymal stem cells (MSCs) are cells with pluripotent characteristics that can be obtained from bone marrow or other sites [33]. The transplanted MSCs release a variety of paracrine factors, including growth factors and extracellular matrix molecules. These factors play a crucial role in regulating the mobilization of endogenous cells, promoting angiogenesis, influencing cellular differentiation, and inducing endogenous stem cells to enhance tissue repair and regeneration [15]. Despite these advantages, unresolved issues such as the potential for tumor formation, low survival rates of transplanted cells, high costs, stability concerns, and stringent regulatory requirements by authorities hinder the widespread clinical application of MSCs [16,17,18,34,35].

Many studies aimed to address the limitations of MSCs. They revealed that the bone regeneration and healing effects are attributed to a paracrine mechanism activated by growth factors released by transplanted MSCs rather than the MSCs themselves. The conditioned medium absorbs these paracrine factors, and studies have indicated their potential for accumulation. Additionally, it has been noted that MSC-CM exhibits significant bone regeneration potential even at low doses, thanks to the collaborative impact of growth factors [15,17,32,36]. MSC-CMs offer advantages such as easier manipulation, storage, and characterization compared with MSCs. They can be sterilized without a loss of efficacy and are readily available for use [19].

This study evaluated the effect of conditioned media derived from mesenchymal stem cells on the bone regeneration ability of allogeneic bone graft material. For this purpose, two 8 mm defects were created in the rabbit’s calvarial bone, and only allogeneic bone graft material was grafted on one side. Allogeneic bone graft material mixed with conditioned media derived from mesenchymal stem cells was grafted on the other side. The bone regeneration effects of the two graft materials were analyzed histologically and histomorphometrically during the healing period of 2 and 8 weeks after the experiment.

Bone grafting experiments were conducted on the rabbit calvaria because the bone formation process in the calvaria and alveolar bone is similar in that it is an intramembranous process [37]. The critical defect size for assessing ossification in rabbit skull models is typically 10 to 15 mm [38,39,40]. Although the 8 mm defect diameter is smaller than the critical size, it is sufficient to evaluate the bone regenerative effect of bone graft material. It is presented as an advantageous model for the comparative evaluation of various bone graft materials, so we used it in this study [40]. The 2-week and 8-week periods were selected in this study because rabbit bone regeneration speed is approximately three times that of human bone regeneration speed. In humans, the time it takes for immature new bone to form after the graft is 6 weeks but, in rabbits, it takes 2 weeks, and the time it takes for bone maturation to be completed is about 6 months after the graft in humans but, in rabbits, it takes 8 weeks [41].

Analyzing this study’s histologic and histomorphometric findings, both the HB and HB+GF groups exhibited new bone formation at 2 and 8 weeks. Notably, in the 8-week group, there was a significant increase in new bone formation compared with the 2-week group, and it was confirmed that new bone formed around human bone particles. When comparing the HB+GF group with the HB group, it was observed that the amount of new bone generated in the HB+GF group was significantly higher in both the 8-week and 2-week groups. The difference was more pronounced in the 8-week group than in the 2-week group. Additionally, in the 2-week group, new bone formation was observed in the center of the defect in the HB+GF group, a phenomenon not observed in the 2-week HB group. Moreover, new bone formation was also confirmed between particles in the HB+GF group.

The resorption levels of the bone graft materials were examined, revealing that, in both groups, greater resorption was observed in the 8-week group compared with the 2-week group. While no significant difference was noted between the HB group and the HB+GF group at the 2-week mark, it was observed that, in the 8-week group, there was a higher resorption in the HB+GF group. The distribution of connective tissue area at the bone graft site did not significantly differ between the 2-week and 8-week groups in both sets. Only in the 2-week group did the HB+GF group exhibit a significantly lower distribution than the HB group.

Bone marrow was observed in the 8-week group in both the HB and HB+GF groups, but it was more prominently observed in the HB+GF group. This result indicates that the mixture of MSC-CM with human bone graft material led to faster and more extensive bone regeneration than the non-mixed counterpart.

Several clinical studies have documented the capacity of MSC-CM to stimulate bone regeneration. Osugi et al. evaluated the extent of new bone formation by transplanting MSC-CM/agarose composite gel in a rat calvarial model, assessing it through micro-CT and histological analyses. At 2, 4, and 8 weeks, the MSC-CM group exhibited a significantly increased area of new bone compared with the control group, with the difference becoming more pronounced, particularly after 8 weeks. Additionally, histological analysis at 8 weeks revealed the observation of a bone bridge in the MSC-CM group, indicating a greater extent of bone regeneration compared with the control group [21]. Katagiri et al. conducted transplantation of β-TCP mixed with MSC-CM and β-TCP mixed with PBS into maxillary sinus defects in rabbits. Histological assessments at 2, 4, and 8 weeks revealed notable findings in the MSC-CM group. Early signs of graft degradation and resorption, coupled with the replacement by newly formed bone, were observed as early as 2 weeks after transplantation. By the 4th week, there was evident development of newly formed bone extending across the entire maxillary sinus floor, accompanied by bone maturation indicators. Conversely, the PBS/b-TCP group displayed minimal graft degradation and replacement with native bone tissue. At 8 weeks, both groups demonstrated newly formed bone covering nearly identical areas, characterized by mature tissue features [20]. In the study by Linero et al., transplantation of a hydrogel containing MSC-CM into a rabbit mandibular bone model was compared with a control group. The radiographic and histological assessments conducted 45 days after surgery revealed a significantly enhanced formation of new bone in the MSC-CM group compared with the control group [22].

This confirms that MSC-CM contains growth factors that regulate the behavior of various other cells associated with bone regeneration. Moreover, it is evident that these growth factors, even at low concentrations, expedite the formation of new bone through various interactions. Osugi et al. also confirmed that MSCs injected into the tail vein of rats migrated to the cranial defect site where MSC-CM was transplanted. Immunohistochemical analysis using the CD44 marker revealed the presence of endogenous MSCs at the junction site of the cranial bone. This result indicates that MSC-CM induces the migration of endogenous MSCs, facilitating a more rapid progression of new bone regeneration [21]. Considering these findings, our study can interpret the growth factor interactions inherent in MSC-CM and the induction of endogenous MSCs to the bone graft site in rabbits as accelerating bone regeneration.

Katagiri et al. analyzed the MSC-CM produced for their research using ELISA. They confirmed higher concentrations of insulin growth factor-1 (IGF-1), vascular endothelial growth factor-A (VEGF), and transforming growth factor-β1 (TGF-β1) compared with other growth factors. Given the known influence of IGF-1, VEGF, and TGF-β1 on bone formation, they hypothesized that these factors might be specific contributors with a stronger impact on the regeneration of new bone. Subsequently, they conducted a comparative study on bone regeneration between MSC-CM, excluding these growth factors, and a cocktail composed solely of them. The results of radiological and histological evaluations showed that the growth factor cocktail exhibited a similar degree of new bone formation as MSC-CM. However, in the case of growth factor-deficient CM, the degree of new bone formation was statistically significantly lower. Through this, they concluded that a combination of IGF-1, VEGF, and TGF-β1, when used together, is effective for bone regeneration even at low concentrations. Additionally, they hypothesized that the combination of IGF-1, VEGF, and TGF-β1 might be ideal for this purpose [23].

The multiplex immunoassay analysis of MSC-CM used in this study revealed different concentrations for the three factors. In the study conducted by Katagiri et al., the concentrations of the three factors were measured as follows: IGF-1 at 1386 ± 465 pg/mL, VEGF at 468.5 ± 109 pg/mL, and TGF-β1 at 339.8 ± 14.4 pg/mL44. In our study, VEGF was not measured, while IGF-1 and TGF-β1 were measured at concentrations of 119.5 pg/mL and 3238.3 pg/mL, respectively. Despite differences in the concentrations of IGF-1 and TGF-β1 and the absence of VEGF measurement, the histological and histomorphometric results of this study revealed that the human bone grafts mixed with MSC-CM formed more new bone at an earlier stage compared with the control group, like the findings in Katagiri et al.’s study [23].

Considering why similar results were obtained despite the difference in concentrations, it is hypothesized that IGF-1 and TGF-β1 may have a more significant impact on bone regeneration than VEGF. IGF-1 is present in bone tissue and is considered to regulate the movement of osteoblasts and mesenchymal stem cells [42,43,44]. Additionally, IGF-1 has been documented to enhance VEGF expression through hypoxia-inducible factor-2a (HIF-2a) [45]. TGF-β1 is known to be a factor that increases bone formation by recruiting osteoprogenitor cells, stimulating their proliferation and differentiation into osteocytes, and regulating the production of extracellular matrix [46,47]. TGF-β1 is also reported to enhance VEGF expression in human cerebral microvascular endothelial cells [48] and increase the amount of available IGF by downregulating the expression of IGF-binding proteins [49]. Based on the information above, it is suggested that even if it contains only IGF-1 and TGF-β1 without VEGF, surrounding endogenous MSCs can express VEGF through IGF-1 and TGF-β1 after migration.

Another factor that may have influenced these results is the use of human bone grafts containing BMP. In the study by Katagiri et al., an absorbable atelocollagen sponge (TERUDERMIS; Olympus Terumo Biomaterials, Tokyo, Japan) that absorbed a cocktail of growth factors was used [23]. BMP is considered to have osteoinductive properties [50], and it is known to induce bone regeneration by promoting the differentiation of mesenchymal cells in the surrounding stromal tissue into osteoblasts. Indeed, there are reports indicating that the presence of BMP in human bone graft materials stimulates the phenotypic transition of undifferentiated mesenchymal progenitor cells into osteoblasts, thereby promoting the formation of new bone [51,52].

One limitation of the present study is the small sample size, which may restrict the statistical power and generalizability of the findings. In addition, bone regeneration was evaluated exclusively through histological and histomorphometric analyses. Although these are well-established techniques, the absence of complementary methods such as micro-computed tomography, immunohistochemistry, or molecular analysis may limit the comprehensive interpretation of the regenerative outcomes. Furthermore, although two growth factors (IGF-1 and TGF-β1) were identified in the MSC-CM based on manufacturer-provided multiplex immunoassay data, a more thorough characterization of other potentially osteogenic cytokines or proteins was not conducted.

These limitations highlight the need for future studies with larger sample sizes, broader molecular profiling of MSC-CM, and multimodal evaluation techniques. In particular, it would be beneficial to conduct follow-up studies employing selected growth factor cocktails or using graft materials with only osteoinductive properties (e.g., xenografts lacking BMP) to further elucidate the specific contributions of MSC-CM to bone regeneration.

## 5. Conclusions

This study evaluated the bone regeneration ability of human bone graft materials mixed with MSC-CM compared with those without MSC-CM in a rabbit calvarial model, using histological and histomorphometric analyses. The experimental group mixed with MSC-CM showed earlier and more substantial new bone formation, indicating enhanced osteogenesis compared with the control group. Consequently, using MSC-CM could lead to earlier and more effective bone regeneration, addressing issues related to cost and safety associated with stem cells.

## Data Availability

The data presented in this study are available on request from the corresponding author.
